# Supplementary material for: Patterns of Intron Gain and Loss in Fungi
Source: PLoS Biol. 2004 Nov 30;2(12):e422. doi: 10.1371/journal.pbio.0020422 (PMC532390; doi:10.1371/journal.pbio.0020422)
Supplement: Table S1 — Also available at http://genes.mit.edu/NielsenEtAl/. (4.3 MB ZIP). [file pbio.0020422.st001.zip › NielsenEtAl/html/1392.html]

AN3147.1.NCU07947.1.MG09396.1.FG01974.1


```
 CLUSTAL W (1.82) Multiple Sequence Alignments - Introns Inserted


Sequence 1: MG09396.1	203 aa
Sequence 2: FG01974.1	206 aa
Sequence 3: AN3147.1	192 aa
Sequence 4: NCU07947.1	206 aa
Alignment Length: 207 aa
Number Identitical Residues: 86 aa
Alignment Score (without introns) 4388


MG09396.1 	MSAVVIPAGGTFVDTLKKSFVDVPIDANNDNAIGTTEFLEAAESLTTIF-~--G1SVAFT
NCU07947.1	MSTGQIPPGGTYLNTLKRSFTDVPVQADNGNAIPTTEFLEAAESLVSIFD~VLG~SAAFS
FG01974.1 	MSDAAYPPGGTIVQSLKRSFVDVPVDAANGDAVSTTEFLEASESLTTIFD1AIG~GMAFG
AN3147.1  	-MPAEIPAGGTWFDTLKRSFDDVPVDAANDNAIATSEFLEAAESLTTLFD1VLG~SAAFS
          	      *.*** .::**:** ***::* *.:*: *:*****:***.::*.   * . ** 

MG09396.1 	PVKNDILGNIK0K-VRDRQLAAPAE~SETIQALVINELKTKKHTAAEGLLWLVR2GLEFM
NCU07947.1	PVKSDMLGNVE0K-IRQRFLAAPTE~SETLQDLVNNEQKAKENKAGQALLWLVR2GLEFT
FG01974.1 	PVKKDILGNVE0K-LRARQAAAPAE~SGTVQDLVRNELKTGKHTATEGCLWLVR2GLDFT
AN3147.1  	PVKSDLTGNIK~VRLSPRSGVAPSN0-------------TKKHVATEGLVWLVR2GLEFT
          	***.*: **::   :  *  .**::              : :: * :. :**** **:* 

MG09396.1 	CIALSQNIAKTSEELADSFRTAYGATLKPHHSFLVKPIFSAAMSAVPYRKDFYAKVGSDQ
NCU07947.1	CKGLANNVAAADQELSTSFRAAYDVTLKPHHSFLIKPIFSAAMSACPYRKDFYTKLGDDQ
FG01974.1 	CQALERNVANSSEELADSFRTAYGNTLKPHHSFVVKPIFSAAMSAVPYRKDFYAKLGENP
AN3147.1  	AKALRHNLDN-GTELSDSFRDAYGKTLKQHHSFMIKPIFSAAMSATPYRKDFYAKLGSDE
          	. .* .*:   . **: *** **. *** ****::********** *******:*:*.: 

MG09396.1 	EKVSTELAKYLASLDKLIGIIKGFQERKEAKW
NCU07947.1	DKVNAQLKEYLAALENFVNILKAFLDSKGIKK
FG01974.1 	QQVEADLQTYLASFTKIVNILKTFINGPEGKW
AN3147.1  	GKVKAALEREVAALEKRVEILKSFQERKEAKW
          	 :*.: *   :*:: : : *:* * :    *
```
